# Total Elbow Arthroplasty for Distal Humerus Fractures in Japanese Elderly Patients: Clinical Outcomes of 23 Cases with Minimum 2-year Follow-up

**DOI:** 10.31662/jmaj.2026-0261

**Published:** 2026-07-24

**Authors:** Yukihiro Kajita, Yohei Harada, Ryosuke Takahashi, Ryosuke Sagami, Yusuke Iwahori

**Affiliations:** 1 Department of Orthopaedic Surgery, Ichinomiya Nishi Hospital, Ichinomiya, Japan; 2 Department of Orthopaedic Surgery, Hiroshima University, Hiroshima, Japan; 3 Department of Orthopaedic Surgery, Asahi Hospital, Kasugai, Japan

**Keywords:** total elbow arthroplasty, distal humerus fracture, Japanese elderly patients, Coonrad-Morrey prosthesis, Nexel elbow, Mayo Elbow Performance Score

## Abstract

**Introduction:**

Open reduction and internal fixation (ORIF) with articular reconstruction is the standard treatment for distal humerus fractures; however, ORIF is challenging in elderly patients with severe comminution and poor bone quality. Although favorable outcomes of total elbow arthroplasty (TEA) have been reported for complex distal humerus fractures deemed unsuitable for ORIF, clinical data in Japanese patients remain limited. This study reports the clinical outcomes of TEA in Japanese patients aged 65 years or older when ORIF was deemed unfeasible.

**Methods:**

We retrospectively reviewed 23 consecutive Japanese patients who underwent primary TEA for complex distal humerus fractures at our institution and had a minimum follow-up of 2 years. The mean age at surgery was 79.3 (standard deviation [SD] 9.0) years (range 65-95 years), and the cohort comprised two men and 21 women. The mean follow-up duration was 40.1 (SD 20.1) months. All procedures were performed via the Campbell approach with ulnar nerve release and anterior transposition. Distal bone fragments were excised, and cemented semiconstrained prostheses were implanted (Coonrad-Morrey: n = 8 [34.8%]; Nexel elbow: n = 15 [65.2%]). Outcome measures included operative time, range of motion (flexion, extension, pronation, and supination), Mayo Elbow Performance Score (MEPS), and postoperative complications.

**Results:**

The mean operative time was 138.9 (SD 19.2) minutes. At final follow-up, the mean range of motion was 125.4° (SD 15.3°) for flexion, −15.3° (SD 16.1°) for extension, 75.9° (SD 4.8°) for pronation, and 74.3° (SD 5.1°) for supination. The mean MEPS was 76.7 (SD 14.4) points. Complications included heterotopic ossification, aseptic loosening of the humeral component, and superficial and deep infections. The overall complication rate was 26.1%, with a reoperation rate of 4.3%.

**Conclusions:**

TEA demonstrated satisfactory functional outcomes and good range of motion in Japanese elderly patients. Complication rates were within acceptable ranges reported in the literature. TEA may be a viable option for complex distal humerus fractures in the Japanese population.

## Introduction

The treatment of distal humerus fractures in elderly patients is challenging due to severe comminution, poor bone quality, and damage to the joint surface. Although open reduction and internal fixation (ORIF) with plates and screws is the standard treatment ^(1), (2)^, osteoporosis and fracture complexity preclude stable fixation, making reliable reconstruction difficult. The bone may not be strong enough to adequately hold the plates and screws, especially when it is severely comminuted ^(3), (4)^. In some cases, it is almost impossible to reduce the comminuted fragments in a way that achieves sufficient healing.

Over the past two decades, total elbow arthroplasty (TEA) has emerged as a potentially viable alternative for elderly patients with distal humerus fractures unsuitable for ORIF. TEA replaces the fractured joint with an artificial joint made of metal and plastic. Several studies have shown that TEA achieved better outcomes compared with ORIF for elderly patients with severe fractures. A randomized trial by McKee et al. ^(5)^ demonstrated superior 2-year functional outcomes with TEA compared with ORIF in 42 patients aged 65 years or older. The study found that patients who underwent TEA achieved better Mayo Elbow Performance Score (MEPS), and 25% of patients initially assigned to undergo ORIF were unable to receive the procedure due to the complexity of their fractures during surgery. These results show that ORIF may not be a feasible option for all elderly patients. Furthermore, a meta-analysis by Githens et al. ^(6)^ reported comparable function and range of motion between TEA and ORIF, with fewer complications and reoperations following TEA.

Long-term follow-up studies, including >12-year data from Dehghan et al. ^(7)^, have shown durable implant survival, with reported complication rates of 10%-24% and reoperation rates of 4%-12%. The study reported >12-year follow-up of 25 patients from McKee’s original cohort, showing durable outcomes after TEA, with only one early revision. All seven surviving patients retained well-functioning implants, and implants remained functional at death in 15 who had deceased, suggesting lifetime durability for most elderly patients. Other recent studies have reported comparable results, with complication rates of 10%-24% and reoperation rates of 4%-12%, supporting TEA as a safe option in this population ^(8), (9), (10)^. These rates are acceptable and show that TEA is a safe treatment option for elderly patients.

Most studies on TEA for distal humerus fractures originate from North America and Europe, with very limited data from Asia, particularly Japan. To date, only a single English-language case report from Japan has been published, which described a special case of elbow injury ^(11)^. Potential differences in body size, bone quality, activity level, and cultural expectations underscore the need for population-specific data to guide treatment decisions and assess the applicability of Western outcomes to Japanese patients. In the absence of such data, surgeons are unable to assess whether TEA offers comparable effectiveness in Japanese patients relative to Western populations.

This study aimed to evaluate the clinical outcomes, complications, and mid- to long-term results of TEA for complex distal humerus fractures in Japanese patients aged ≥65 years and to compare these outcomes with those reported internationally. We hypothesized that TEA would yield outcomes comparable to those reported in other populations.

## Materials and Methods

### Study design and ethical considerations

This study was a retrospective review of consecutive patients who underwent primary TEA for complex distal humerus fractures not amenable to internal fixation at our institution between 2009 and 2023. This research was approved by the Institutional Review Board of Ichinomiya Nishi Hospital (approval number: 2025101). The study was performed in accordance with the ethical standards outlined in the 1964 Declaration of Helsinki and its subsequent amendments. Written informed consent was obtained from all patients. All procedures were performed by experienced upper extremity surgeons via a standardized surgical technique.

### Patient inclusion and exclusion criteria

Patients were eligible if they met the following criteria: (1) age 65 years or older; (2) acute distal humerus fracture or failed internal fixation requiring conversion to TEA; (3) fracture deemed not amenable to stable internal fixation due to severe comminution, poor bone quality, or both; and (4) minimum clinical follow-up of 24 months. Patients were excluded if they had undergone revision TEA, had incomplete clinical records that prevented adequate evaluation, or required conversion to TEA following failed ORIF. Additionally, while patients undergoing TEA for chronic inflammatory arthropathy or joint destruction as the primary indication were excluded, those who sustained an acute distal humerus fracture as the primary diagnosis and had a relevant medical history of controlled rheumatoid arthritis (RA) were eligible for inclusion.

During the study period between 2009 and 2023, a total of 25 consecutive patients underwent TEA for distal humerus fractures at our institution. Among them, two patients who underwent conversion TEA for failed internal fixation were excluded to ensure cohort homogeneity. The remaining 23 patients who underwent primary TEA for acute fractures were included in the final analysis. Notably, no patients were lost to follow-up, and complete clinical and radiographic data were available for all 23 patients (100% follow-up rate) at the minimum 24-month follow-up evaluation.

### Implant selection

The selection of the prosthesis type was determined based on the chronological period of surgery at our institution. The Coonrad-Morrey prosthesis (Zimmer Biomet, Warsaw, IN, USA) was routinely used from the beginning of the study period through February 2016. Subsequently, the newer-generation Nexel prosthesis (Zimmer Biomet, Warsaw, IN, USA) was introduced and used from March 2016 onward.

### Clinical evaluation

Clinical outcomes were assessed using the MEPS and active range of motion at final follow-up. The MEPS is a 100-point scale that includes pain, range of motion, stability, and daily function. Active range of motion was measured with a goniometer for flexion, extension, pronation, and supination. All measurements were performed by trained physical therapists who were not involved in the surgical procedures.

Complications were recorded from medical records and included heterotopic ossification, aseptic loosening, superficial infection, deep infection, nerve injury, and periprosthetic fracture. Infections were classified as superficial if they involved only the skin and subcutaneous tissue without implant involvement and as deep if they involved the joint space or required implant removal.

Furthermore, to investigate the potential factors influencing these clinical and safety outcomes, subgroup analyses were performed. The clinical scores, active range of motion, and complication rates were comparatively evaluated between patients with RA and those without RA. Additionally, a descriptive subgroup comparison of outcomes was conducted between the two different prosthesis types (the Coonrad-Morrey prosthesis and the Nexel prosthesis) used in this cohort.

### Radiographic evaluation

Standard anteroposterior and lateral radiographs of the elbow were obtained immediately after surgery and at the most recent follow-up. Radiographs were reviewed by two independent observers for evidence of aseptic loosening, periprosthetic lucency, heterotopic ossification, or component migration. Aseptic loosening was defined as gross migration or change in implant position or the presence of continuous radiolucent lines greater than 2 mm at the bone-cement interface surrounding the component.

### Surgical technique

All procedures were conducted under general anesthesia with the patient placed in the lateral decubitus position. A posterior skin incision and a Campbell approach were used in all cases. The Campbell approach involves reflecting the triceps mechanism medially and laterally without detaching it from the olecranon, which preserves extensor function. The ulnar nerve was identified and routinely released from the cubital tunnel and transposed anteriorly to prevent postoperative nerve compression.

After exposure of the fracture site, all distal bone fragments that could not contribute to stable fixation were removed. The medullary canals of the humerus and ulna were prepared according to the manufacturer’s guidelines for each prosthesis type. Antibiotic-loaded polymethylmethacrylate cement was used to secure both the humeral and ulnar components, following the standard application procedure.

After component implantation and reduction of the joint, the stability and range of motion were checked. The triceps mechanism was repaired to its normal anatomical position through the preserved insertion on the olecranon. Layered closure was performed, and a closed suction drain was placed in all cases. The drain was maintained until the first postoperative dressing change at 2 days after surgery to reduce hematoma formation.

### Postoperative management

Postoperatively, all elbows were immobilized in extension using a posterior splint for 2 days. The drain was removed at the time of the first dressing change. Gentle active and passive range-of-motion exercises were initiated on postoperative day three under the supervision of a physical therapist. Progressive strengthening exercises began at 6 weeks postoperatively. All patients were instructed to limit loads to 5 kg for single lifts and 1 kg for repetitive activities to minimize stress on the implant.

### Statistical analysis

Descriptive statistics were used to summarize demographic data, clinical outcomes, and complication rates. Continuous variables were expressed as mean and standard deviation (SD) (range) and categorical variables as counts and percentages. Statistical analyses were performed using Welch’s t test or the National Institutes of Health–recognized Mann-Whitney U test for continuous variables to compare the functional outcomes between subgroups. Fisher’s exact test was used to evaluate the difference in complication rates between patients with RA and those without RA. Statistical significance was set at p <0.05.

## Results

### Patient characteristics

A total of 23 patients (23 elbows) met the inclusion criteria. The cohort comprised two men and 21 women, with a mean age of 79.3 (SD 9.0) years (range 65-95 years). The mean follow-up period was 40.1 (SD 20.1) months (range 24-96 months). The affected side was the dominant extremity in 14 elbows and the nondominant extremity in nine elbows. Nine patients had coexisting RA. The mean duration from injury to surgery was 13.1 (SD 9.6) days (range 2-36 days). According to the AO Foundation/Orthopaedic Trauma Association (AO/OTA) classification system, the fracture types included seven cases of type 13-A3, three of type 13-C1, four of type 13-C2, and nine of type 13-C3.

### Surgical outcomes

The mean operative time for all procedures was 138.9 (SD 19.2) minutes (range 95-180 minutes). No intraoperative complications occurred during any of the procedures.

### Clinical outcomes

The clinical outcomes are shown in Table 1. At final follow-up, the mean MEPS was 76.7 (SD 14.4) points (range 45-100 points). According to the MEPS classification, three elbows (13%) were rated as excellent, 18 (78.3%) as good, 1 (4.3%) as fair, and 1 (4.3%) as poor.

**Table 1.** Clinical Outcomes at Final Follow-Up (n = 23).

Parameters	Values
MEPS (points), mean (SD; range)	76.7 (14.4; 45 to 100)
Excellent (90-100), n (%)	3 (13.0)
Good (75-89), n (%)	18 (78.3)
Fair (60-74), n (%)	1 (4.3)
Poor (<60), n (%)	1 (4.3)
ROM (degrees), mean (SD; range)
Flexion	125.4 (15.3; 90 to 150)
Extension	−16.1 (15.3; 0 to −45)
Pronation	75.9 (4.8; 65 to 85)
Supination	74.3 (5.1; 65 to 85)

MEPS: Mayo Elbow Performance Score; ROM: range of motion; SD: standard deviation.

The mean active range of motion at final follow-up was as follows: flexion 125.4° (SD 15.3°) (range 90°-150°), extension −16.1° (SD 15.3°) (range 0° to −45°), pronation 75.9° (SD 4.8°) (range 65°-85°), and supination 74.3° (SD 5.1°) (range 65°-85°). The mean flexion-extension arc was 108.7° (SD 26.2°), and the mean pronation-supination arc was 150.2° (SD 7.8°).

### Complications

The overall complication rate was 26.1% (6 of 23 patients). The details of complications are shown in Table 2. Complications included heterotopic ossification in one patient (4.3%), aseptic loosening of the humeral component in one patient (4.3%), superficial infection in three patients (13.0%), and deep infection in one patient (4.3%).

**Table 2.** Complications and Their Clinical Course (n=23).

Complications	Patients, n (%)	Management	Outcome	RA status
Heterotopic ossification	1 (4.3)	Conservative	No functional limitation	+
Aseptic loosening (humeral component)	1 (4.3)	Conservative observation	Stable, no pain, good function	+
Superficial infection	3 (13.0)	Oral antibiotics and wound care	Complete healing, no recurrence	All +
Deep infection (MRSA)	1 (4.3)	Implant removal at 5 months	Infection eradicated, flail elbow	+

Note: All six complications, including heterotopic ossification, occurred exclusively in patients with underlying RA. MRSA: methicillin-resistant *Staphylococcus aureus*; RA: rheumatoid arthritis.

The patient with heterotopic ossification showed mild bone formation around the elbow joint; however, the ossification did not affect function and required no treatment. The patient with aseptic loosening of the humeral component was identified on routine radiographs at 36 months postoperatively. This patient had no pain and good function, and the loosening was managed conservatively without revision surgery throughout the follow-up period.

Three patients developed superficial wound infections within the first 2 weeks after surgery. All three were successfully treated with oral antibiotics and local wound care without requiring reoperation. The wounds healed completely, and there was no recurrence of infection.

One patient developed deep infection at 4 months postoperatively. The causative organism was methicillin-resistant *Staphylococcus aureus* (MRSA). Despite initial treatment with intravenous antibiotics, the infection could not be controlled. The patient underwent implant removal at 5 months postoperatively. After a 6-week course of intravenous antibiotic therapy, the infection was successfully eradicated. However, the patient declined further reconstruction and was managed with a flail elbow. This was the only case that required reoperation in this series, giving a reoperation rate of 4.3% (1 of 23 patients).

No patients experienced nerve injury, periprosthetic fracture, or mechanical failure of the implant during the follow-up period. No patients required revision surgery for aseptic loosening or mechanical failure.

### Subgroup analyses according to RA and prosthesis type

Regarding clinical outcomes at the final follow-up, there were no statistically significant differences between the RA and non-RA groups in terms of the mean MEPS (71.1 [SD 20.1] points vs. 80.4 [SD 8.2] points; p = 0.220), flexion (121.1° [SD 17.1°] vs. 128.2° [SD 14.6°]; p = 0.320), or extension (−20.6° [SD 19.1°] vs. −13.2° [SD 12.0°]; p = 0.324). However, the complication rate was significantly higher in the RA group compared to the non-RA group (66.7% [6/9] vs. 0.0% [0/14]; Fisher’s exact test, p = 0.00083) (Table 3).

**Table 3.** Comparison of Clinical Outcomes and Complication Rates between Patients with RA and Those without RA.

Parameters	RA group (n = 9)	Non-RA group (n = 14)	p-Value
MEPS (points), mean (SD)	71.1 (20.1)	80.4 (8.2)	0.220
ROM (degrees), mean (SD)			
Flexion	121.1 (17.1)	128.2 (14.6)	0.320
Extension	−20.6 (19.1)	−13.2 (12.0)	0.324
Pronation	74.4 (3.9)	76.8 (5.4)	0.243
Supination	75.7 (4.6)	73.6 (5.7)	0.372
Complication rate, % (n)	66.7 (6)	0 (0)	0.0008

MEPS: Mayo Elbow Performance Score; RA: rheumatoid arthritis; ROM: range of motion; SD: standard deviation.

Regarding the type of prosthesis, eight patients received the Coonrad-Morrey prosthesis and 15 received the Nexel prosthesis. In the Coonrad-Morrey group, the mean MEPS was 70.6 (SD 21.1) points, and the mean range of motion was 126.8° (SD 16.9°) for flexion, −21.9° (SD 19.3°) for extension, 74.4° (SD 3.2°) for pronation, and 76.3° (SD 4.4°) for supination. In the Nexel group, the mean MEPS was 80.0 (SD 8.5) points, and the mean range of motion was 124.7° (SD 15.5°) for flexion, −13.0° (SD 12.2°) for extension, 76.7° (SD 5.6°) for pronation, and 73.3° (5.6°) for supination. Complications occurred in three patients (37.5%) in the Coonrad-Morrey group and in three patients (20.0%) in the Nexel group (Table 4).

**Table 4.** Descriptive Subgroup Analysis of Clinical Outcomes and Complications between the Two Prosthesis Types.

Parameters	Coonrad-Morrey group (n = 8)	Nexel group (n = 15)	p-Value
MEPS (points), mean (SD)	70.6 (21.1)	80.0 (8.5)	0.262
ROM (degrees), mean (SD)			
Flexion	126.9 (16.9)	124.7 (15.5)	0.763
Extension	−21.9 (19.3)	−13.0 (12.2)	0.264
Pronation	74.4 (3.9)	76.8 (5.4)	0.243
Supination	76.3 (4.4)	73.3 (5.6)	0.187
Complication rate, % (n)	37.5 (3)	20.0 (3)	0.617

MEPS: Mayo Elbow Performance Score; ROM: range of motion; SD: standard deviation.

## Discussion

This study reports the clinical outcomes of TEA for complex distal humerus fractures in 23 Japanese elderly patients with a mean follow-up of 40.1 months. To our knowledge, this is one of the few studies from Asia, and the first from Japan, to evaluate the midterm outcomes of TEA for distal humerus fractures with a minimum 2-year follow-up. Our results showed satisfactory functional outcomes with a mean MEPS of 76.7 points, good range of motion, and a low reoperation rate of 4.3%.

One of the main advantages of TEA over ORIF in elderly patients with complex distal humerus fractures is the early recovery of elbow motion. As demonstrated in our representative case (Figure 1), the patient achieved a satisfactory range of motion as early as 3 months postoperatively, and this functional improvement was well maintained until the final follow-up. In contrast, patients treated with ORIF often require prolonged protection of motion for fracture healing, typically 3-6 months, which may predispose elderly patients to stiffness and functional loss. Consistent with prior reports, including the study by McKee et al. ^(5)^, who demonstrated superior 2-year functional outcomes with TEA, our findings show that even very elderly patients (mean age 79.3 years) can achieve favorable functional recovery following TEA.

**Figure 1.**
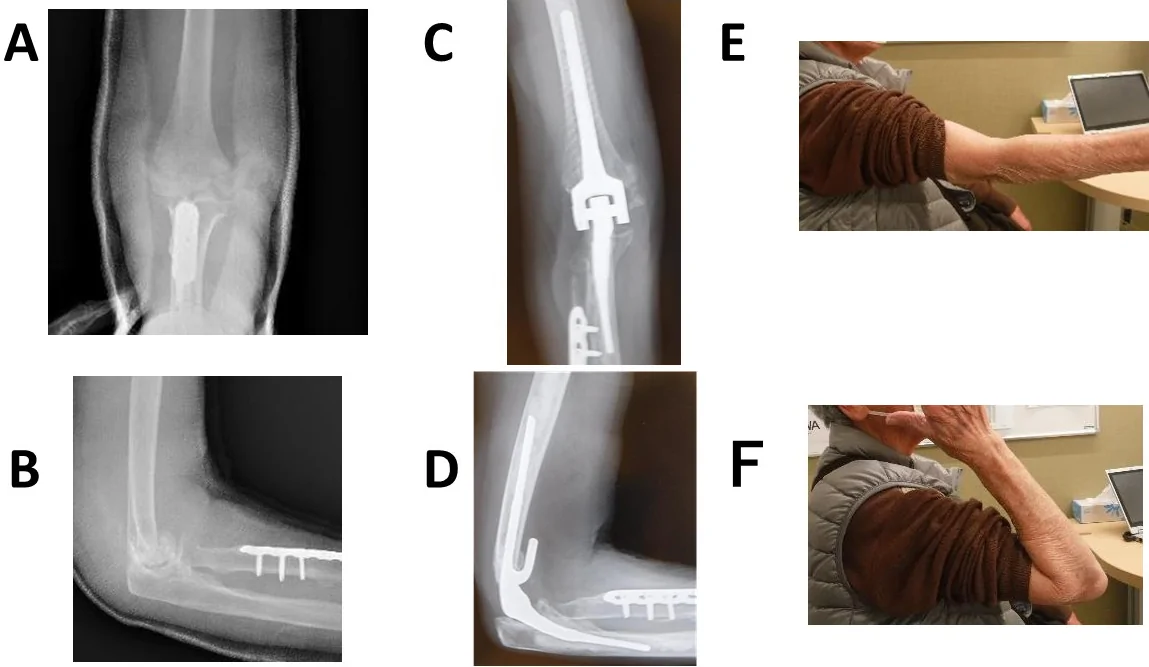
A 92-year-old woman sustained a distal humerus fracture after a fall. She had been living independently before the injury. (A, B) Preoperative anteroposterior and lateral radiographs showing a comminuted distal humerus fracture not amenable to stable internal fixation. (C, D) Postoperative radiographs showing good component position and stable fixation. (E, F) Clinical photographs at 3 months postoperatively showing elbow extension and flexion. The patient achieved a Mayo Elbow Performance Score of 85 points at final follow-up with excellent functional recovery and return to independent living.

This is consistent with prior reports: Somerson et al. ^(12)^ identified RA as an independent risk factor for periprosthetic joint infection after TEA, and a meta-analysis by Wang et al. ^(13)^ reported a 31.6% complication rate in RA patients. Immunosuppressive therapy, chronic inflammation, and compromised bone quality in RA may contribute to impaired healing and increased infection risk. One of the most important findings in our study is that all six complications occurred in patients with coexisting RA. Complications developed in 6 of 9 (67%) RA patients, whereas no complications were observed among the 14 patients without RA, suggesting RA as a strong risk factor for adverse outcomes after TEA in fracture cases. Previous studies have reported similar findings. Somerson et al. ^(12)^ identified RA as a significant independent risk factor for periprosthetic joint infection after TEA, with an odds ratio of 3.31. A systematic review and meta-analysis by Wang et al. ^(13)^ comparing outcomes of TEA in RA versus posttraumatic conditions found higher complication rates in RA patients, supporting our findings. Patients with RA often use immunosuppressive medications such as methotrexate or biological agents, which can impair wound healing and increase infection risk. Additionally, RA itself causes chronic inflammation and poor bone quality, which may affect implant fixation and healing.

Our deep infection case occurred in a RA patient who was taking immunosuppressive medication. This patient developed MRSA infection at 4 months postoperatively and required implant removal. Although TEA is an effective treatment option for elderly patients with complex distal humerus fractures, complications such as deep infection can have severe consequences. When deep infection occurs, salvage options are very limited and often result in poor function. In our case, the patient declined further reconstruction after implant removal and was left with a flail elbow, underscoring the difficulty of managing such complications. Therefore, careful patient selection is essential, especially in patients with RA who are at higher risk. Surgeons should thoroughly discuss the increased risk of complications with these patients before surgery and carefully weigh the benefits against the potentially dire consequences of infection.

We compared our results with previous studies that had a minimum 24-month follow-up (Table 5). Our reoperation rate of 4.3% was the lowest reported, including rates from a recent French study by Palladino et al. ^(8)^ (5%) and a Spanish study by Lopiz et al. ^(9)^ (8%). Although our overall complication rate (26.1%) was higher than those reported by Palladino et al. ^(8)^ (8%) and Ducrot et al. ^(10)^ (10%), this likely reflects the high proportion of patients with RA in our cohort (39.1%; 9 of 23 patients). When considering the distribution of these adverse events, the complication rate in the RA subgroup was 66.7% (6 of 9 patients), whereas no complications occurred in the non-RA subgroup (0.0%; 0 of 14 patients), demonstrating a highly significant statistical difference. These findings suggest that TEA is a very safe procedure with minimal serious complications in patients without RA. Our mean MEPS of 76.7 points was lower than that in some European studies (Palladino et al. ^(8)^: 90 points, Lopiz et al. ^(9)^: 88 points, and Ducrot et al. ^(10)^: 86 points). However, our patient population was older and included more RA patients. Despite these challenging patient factors, a mean score of 76.7 points still represents good functional outcomes according to the MEPS classification system.

**Table 5.** Comparison of Clinical Outcomes with Previous Studies.

Study	Country	Patients (n)	Mean age (years)	Mean follow-up (months)	Mean MEPS (points)	Complication rate (%)	Reoperation rate (%)
Current study	Japan	23	79.3	40.1	76.7	26.1	4.3
Palladino et al.^(8)^, 2024	France	60	80.0	40.8	90.0	8.0	5.0
Lopiz et al.^(9)^, 2021	Spain	12	81.0	60.0	88.0	25.0	8.0
Ducrot et al.^(10)^, 2013	France	87	79.0	37.5	86.0	10.0	10.0

MEPS: Mayo Elbow Performance Score.

Clinical data regarding TEA for distal humerus fractures remain sparse in Asia. Baik et al. ^(14)^ reported favorable short-term outcomes of TEA compared with ORIF in an older Korean population. Furthermore, a systematic review by Kholinne et al. ^(15)^ highlighted the midterm outcomes of a small cohort from India (21 patients; mean follow-up: 55.5 months). However, most previous Asian studies primarily used older-generation implants, such as the Coonrad-Morrey prosthesis.

The clinical significance of our study lies in providing the largest Japanese cohort (23 patients) with a mean follow-up of 40.1 months, particularly focusing on the Nexel prosthesis (65.2%). Although some Western literature has raised concerns regarding early failure rates of the Nexel design, our midterm results demonstrated a remarkably low reoperation rate (4.3%) and excellent implant survival in low-demand Japanese elderly patients. Therefore, our findings complement the scarce Asian literature and offer crucial geographic-specific data for upper extremity surgeons in Asia.

We used two types of prostheses in this study: Coonrad-Morrey in eight elbows (34.8%) and Nexel in 15 elbows (65.2%). Recent literature on the Nexel prosthesis has reported concerning early outcomes. For example, Morrey et al. ^(16)^ reported an unexpectedly high early failure rate, with a 34% revision rate at an average of 2.2 years, primarily due to aseptic loosening. Similarly, Siala et al. ^(17)^ reported abnormally high complication and revision rates in a series of nine Nexel TEA procedures, particularly related to humeral component issues.

In our study, we observed only one case of aseptic loosening in a Nexel prosthesis patient, which was managed conservatively without revision, and no mechanical failures or periprosthetic fractures occurred. Several clinical factors might explain this discrepancy. First, our cohort consisted of older patients (mean age: 79.3 years) who typically have lower activity demands compared to younger populations. Second, all our cases were indicated for acute trauma rather than chronic inflammatory conditions such as RA, which might have provided relatively better local bone quality for cement fixation. Finally, we strictly instructed patients to limit loads to 5 kg for a single lift and 1 kg for repetitive activities, potentially protecting the implant from excessive mechanical stress.

Although the Nexel prosthesis yielded acceptable midterm outcomes in our specific cohort of low-demand elderly patients, we emphasize that our findings are based on a limited number of cases and a midterm follow-up period. Given the severe mechanical failures and high complication rates reported in other studies, continuous and long-term careful monitoring remains essential to determine whether these satisfactory outcomes can be maintained over time.

In our study, the clinical outcomes and complication rates were described separately for each prosthesis type. However, because the Coonrad-Morrey and Nexel prostheses were introduced in different sequential time periods at our institution, the Coonrad-Morrey group essentially serves as a historical control. Given the inherent historical bias and chronological differences in the surgeon’s learning curve, a formal statistical comparison between the two implant groups was intentionally avoided to prevent biased conclusions. Nevertheless, the descriptive data suggested that both implants provided acceptable functional recovery for complex distal humerus fractures in elderly patients.

This study has several limitations. First, this was a retrospective study without a control group. We did not compare TEA directly with ORIF in our patient population. However, all our patients had fractures that were determined unsuitable for ORIF; therefore, a randomized comparison would not have been ethical. Second, our follow-up period of 40.1 months represents midterm results. Longer follow-up is needed to determine the durability of these implants, especially for the Nexel prosthesis. Third, our sample size of 23 patients was relatively small, although this represents the largest Asian series reported. Fourth, we did not collect preoperative functional scores; therefore, we cannot report on the pre- to postsurgical improvements of this study. However, as all patients presented with acute fractures, it is reasonable to infer that they exhibited normal or near-normal function prior to the injury. Finally, due to the retrospective nature of this study, detailed clinical data regarding RA characteristics, such as precise disease duration, baseline disease activity scores, and historical stage/class, were not fully available because the included RA patients had their medical treatments managed and controlled at external rheumatology clinics.

Based on our findings, we can make several recommendations. First, TEA is an effective treatment option for elderly Japanese patients with complex distal humerus fractures that cannot be fixed with plates and screws. The procedure provides good functional outcomes and early return to activities. Second, RA is a strong risk factor for complications after TEA. Surgeons should be very careful when considering TEA for RA patients with acute fractures and should discuss the high complication risk with these patients. Third, although TEA is generally effective, when serious complications such as deep infection occur, salvage is very difficult and can result in poor outcomes. Therefore, careful patient selection and thorough informed consent are essential. Fourth, the Nexel prosthesis can perform well in elderly trauma patients, despite considering reports on other patient populations. Finally, strict activity restrictions (limiting lifting to 5 kg) should be enforced to protect the implant.

### Conclusions

TEA for complex distal humerus fractures in Japanese elderly patients achieved satisfactory functional outcomes with a mean MEPS of 76.7 points and a low reoperation rate of 4.3%. This is one of the few studies from Asia, and the first from Japan, to evaluate the midterm outcomes of TEA for distal humerus fractures with a minimum 2-year follow-up. Although TEA is a reliable treatment option for elderly Japanese patients, serious complications such as deep infection can have devastating consequences. Therefore, careful patient selection and thorough informed consent are essential.

## Article Information

### Author Contributions

Yukihiro Kajita: conceptualization, data curation, formal analysis, and writing – original draft; Yohei Harada: investigation and data curation; Ryosuke Takahashi: investigation and data curation; Ryosuke Sagami: investigation and data curation; and Yusuke Iwahori: investigation and data curation. All authors have read and approved the final version of the manuscript.

### Conflicts of Interest

None

### Ethics Approval

This study was approved by the Institutional Review Board of Ichinomiya Nishi Hospital (approval number: 2025101).
